# Notes and Notices

**Published:** 1897-05

**Authors:** 


### NOTES AND NOTICES.

Dr. F. H. Lutze has removed from 271 South Fifth Street to 212 Keap Street, Brooklyn, New York.

Dr. Maro F. Underwood has removed from San Francisco to 602 Telegraph Avenue, Oakland, California, his office and residence being combined.

International Hahnemannian Association.—Do not forget the Bureau of Surgery, and the June meeting of the I. H. A. at Niagara.

Cases you have "saved from the surgeon's knife" are most acceptable, but those requiring operation will be gladly received.

Thos. M. Dillingham, M. D.,
*Chairman, Bureau of Surgery.*

8 West 49th Street, New York City.

The Society of Homeopathicians.—The next annual meeting of the Society of Homeopathicians will be held at "The New Mathewson," Narragansett Pier, R. I., June 22d, 23d, 24th, and 25th.

Rates, $3 per day for room for one; $2.50 each, per day, for two in a room.

This will be an important meeting and early notice is sent so that all can make arrangements to be present.

Dr. Stuart Close, Chairman of Bureau of Homeopathic Philosophy, 641 Willoughby Avenue, Brooklyn, N. Y.

Dr. Olin M. Drake, Chairman of Bureau of Clinical Medicine, 70 Huntington Avenue, Boston, Mass.

S. A. Kimball, *Secretary.*

124 Commonwealth Avenue, Boston, Mass.

What, a Polar Bear?—A Frenchman went to an American and said to him: "What, a Polar Bear?"

The American answered: "Do you mean, what does a polar bear do? Um, well, I don't know. Oh, why, he sits on the ice."

"Sits on ze ice!"

"Yes," said the American, "there is nothing else to sit on, you know."

"Vell, vat he do, too?"

"What does he also do? Why, he eats fish."

"Eats ze fish! Sits on ze ice, and eats ze fish! Then I not accept."

"You don't accept! Why, what do you mean, you don't accept?"

"Oh, non, non, I does not accept. I was invite to be polar bear (pall bearer) to a funeral!"—*Bachelor of Arts.*

